# Biopterin Metabolism and eNOS Expression during Hypoxic Pulmonary Hypertension in Mice

**DOI:** 10.1371/journal.pone.0082594

**Published:** 2013-11-27

**Authors:** Mathilde Dubois, Estelle Delannoy, Lucie Duluc, Ellen Closs, Huige Li, Christian Toussaint, Alain-Pierre Gadeau, Axel Gödecke, Véronique Freund-Michel, Arnaud Courtois, Roger Marthan, Jean-Pierre Savineau, Bernard Muller

**Affiliations:** 1 University Bordeaux, Centre de recherche Cardio-Thoracique de Bordeaux, U1045, Bordeaux, France; 2 INSERM, Centre de recherche Cardio-Thoracique de Bordeaux, U1045, Bordeaux, France; 3 CHU de, Bordeaux, Bordeaux, France; 4 Department of Pharmacology, Johannes Gutenberg University Medical Center, Mainz, Germany; 5 University Bordeaux, Bordeaux, France; 6 INSERM, Adaptation cardiovasculaire à l'ischémie, U1034, Pessac, France; 7 Institute of Cardiovascular Physiology, Heinrich-Heine University, Düsseldorf, Germany; University of Kentucky, United States of America

## Abstract

Tetrahydrobiopterin (BH_4_), which fosters the formation of and stabilizes endothelial NO synthase (eNOS) as an active dimer, tightly regulates eNOS coupling / uncoupling. Moreover, studies conducted in genetically-modified models demonstrate that BH_4_ pulmonary deficiency is a key determinant in the pathogenesis of pulmonary hypertension. The present study thus investigates biopterin metabolism and eNOS expression, as well as the effect of sepiapterin (a precursor of BH_4_) and eNOS gene deletion, in a mice model of hypoxic pulmonary hypertension. In lungs, chronic hypoxia increased BH_4_ levels and eNOS expression, without modifying dihydrobiopterin (BH_2_, the oxidation product of BH_4_) levels, GTP cyclohydrolase-1 or dihydrofolate reductase expression (two key enzymes regulating BH_4_ availability). In intrapulmonary arteries, chronic hypoxia also increased expression of eNOS, but did not induce destabilisation of eNOS dimers into monomers. In hypoxic mice, sepiapterin prevented increase in right ventricular systolic pressure and right ventricular hypertrophy, whereas it modified neither remodelling nor alteration in vasomotor responses (hyper-responsiveness to phenylephrine, decrease in endothelium-dependent relaxation to acetylcholine) in intrapulmonary arteries. Finally, deletion of eNOS gene partially prevented hypoxia-induced increase in right ventricular systolic pressure, right ventricular hypertrophy and remodelling of intrapulmonary arteries. Collectively, these data demonstrate the absence of BH_4_/BH_2_ changes and eNOS dimer destabilisation, which may induce eNOS uncoupling during hypoxia-induced pulmonary hypertension. Thus, even though eNOS gene deletion and sepiapterin treatment exert protective effects on hypoxia-induced pulmonary vascular remodelling, increase on right ventricular pressure and / or right ventricular hypertrophy, these effects appear unrelated to biopterin-dependent eNOS uncoupling within pulmonary vasculature of hypoxic wild-type mice.

## Introduction

Pulmonary hypertension is characterized by functional (vasoconstriction, endothelial dysfunction) and structural (remodelling) alterations in the pulmonary vasculature [1-3], leading to right ventricular hypertrophy, right ventricular failure and ultimately death. The underlying mechanisms include alterations in local production and effects of vasoactive factors, but remain yet not fully understood.

Endothelial NO synthase (eNOS)-derived nitric oxide (NO) exerts vasculoprotective effects [4]. Tetrahydrobiopterin (BH_4_) is a cofactor of eNOS, which fosters dimer formation and stabilizes the enzyme as an active dimer, allowing optimal oxidation of L-arginine into NO [5-7]. A decrease in BH_4_ availability results in eNOS uncoupling, a dysfunctional state in which, following dimer destabilisation, eNOS monomers produce reactive oxygen species (ROS) rather than NO. This contributes to vascular oxidative stress and dysfunctions in many cardiovascular diseases [5-7]. BH_4_ bioavailability depends on a balance between *de novo* synthesis by GTP cyclohydrolase-1 (GTPCH-1, the first and limiting step for BH_4_ biosynthesis from GTP), loss of BH_4_ (due to oxidation of BH_4_ into the dihydrobiopterin BH_2_) and recycling of BH_2_ into BH_4_ by dihydrofolate reductase (DHFR) [7]. Even in the absence of BH_4_ deficiency, elevated levels of BH_2_ can compete with BH_4_ for binding to eNOS, resulting in eNOS uncoupling [8,9]. Rather than the absolute BH_4_ concentration, both eNOS / BH_4_ stoicheiometry and biopterin redox status appear as key determinants of eNOS uncoupling [7,10]. 

Local BH_4_ availability is crucial in maintaining pulmonary vascular homeostasis. Indeed, increased BH_4_ synthesis prevents pulmonary hypertension [11]. Conversely, BH_4_ deficiency elevates pulmonary vascular tone by decreasing eNOS activity and NO bioactivity, thus promoting pulmonary vascular remodelling, pulmonary hypertension and right ventricular hypertrophy [11,12]. These data were obtained in mice models, in which tissue BH_4_ levels were either decreased by reduced expression of GTPCH-1, or increased by targeted overexpression of GTPCH-1. Only few studies have investigated whether alterations in biopterin metabolism occur in pathophysiological relevant models of pulmonary hypertension. In a lamb model of persistent pulmonary hypertension of the newborn, an increase in oxidative stress due to uncoupled eNOS and elevated levels of BH_2_ likely contributes to impaired NO-dependent pulmonary vasodilatation [13,14]. 

In the present study, we analysed biopterin metabolism and eNOS expression in a model of hypoxia-induced pulmonary hypertension. This model is clinically relevant, since chronic hypoxia is one of the major causes of sustained pulmonary hypertension in patients with advanced chronic obstructive pulmonary disease [15]. In mice exposed to chronic hypoxia, in which ROS are elevated in the pulmonary vasculature [16,17], we investigated lung levels of BH_4_ and BH_2_, expression of eNOS (including dimer / monomer ratio), GTPCH-1 and DHFR in lung and/or intrapulmonary arteries, during hypoxic challenge. The effect of sepiapterin (a precursor of BH_4_ *via* DHFR [7]) and eNOS gene deletion on pulmonary vasomotor responses, pulmonary vascular remodelling, right ventricular systolic pressure and hypertrophy was also evaluated.

## Materials and Methods

### Animals

This investigation conformed to the Guide for the Care and Use of Laboratory Animals (NIH Publication No. 85-23, revised 1996). Agreement (number A 33409) was obtained from French authorities and all the protocols used were approved by our local ethics committee named Comité d'éthique régional d'Aquitaine (protocol number: AP 2/11/2005). Experiments were performed in 10 weeks old (20–25 g) male C57Bl/6 wild type (WT) mice (Elevage Janvier, Le Genest St Isle, France) and male C57Bl/6 *eNOS* ^*-/-*^ mice [18,19]. Some WT and *eNOS* ^*-/-*^ mice were housed in a hypobaric chamber at 380 mmHg for 10, 21 or 40 days. The chamber was opened every other day for 30 min for animal care and cleaning. Control mice (normoxic group) were housed in ambient atmospheric conditions. For some experiments, sepiapterin (30 mg / kg) was administered orally by gavage, one day before exposure to hypoxia and every other day during hypoxia, at each chamber opening. Consequently, the dose of sepiapterin was adapted from previous studies [20,21].

After cervical dislocation, the heart and lungs were removed and placed in cold Krebs solution (composition in mM: NaCl 119; KCl 4.7; CaCl_2_ 1.5; MgSO_4_ 1.17; KH_2_PO_4_ 1.18; NaHCO_3_ 25; and glucose 5.5).

### Measurement of pulmonary BH_4_ and BH_2_

Mice lungs were homogenized in ice-cold lysis buffer (0.1 mM Tris-HCl, pH 7.8, containing 5 mM ethylenediamine tetraacetic acid, 0.3 mM KCl, 5 mM 1,4-dithioerythritol, 0.5 mM Pefabloc, and 0.01% saponin). Samples were oxidized under either acidic conditions (with 0.2 M HCl containing 50 mM I_2_) or alkaline conditions (with 0.2 M NaOH containing 50 mM I_2_). The biopterin content was assessed by HPLC with fluorescence detection (350-nm excitation, 450-nm emission). The BH_4_ concentration was calculated by subtracting the biopterin peak resulting from alkaline oxidation (accounting for BH_2_) from the biopterin peak resulting from acidic oxidation (accounting for both BH_2_ and BH_4_) [22]. Biopterin concentrations were expressed as picomoles per milligram of protein.

### Expression of GTPCH-1, DHFR, total eNOS and eNOS dimer / monomer

For Western-blotting, tissues were homogenized in ice-cold lysis buffer (50 mM Tris, pH 7.5, 150 mM NaCl, 0.1% SDS, 0.5% deoxycholate, 1% Nonidet P-40) containing protease inhibitors and 1 mM phenylmethylsulfonyl fluoride. Homogenates (30 µg of proteins) were separated by electrophoresis on SDS-PAGE (at 4°C for eNOS dimer / monomer analysis; [23]) and transferred to PVDF membrane. After blocking, membranes were incubated with antibodies against eNOS, DHFR, GTPCH-1, or β-actin and goat anti mouse IgG peroxidase conjugated antibody. Protein bands were visualized by chemiluminescence and quantified using Image J software (National Institutes of Health, Bethesda, MA, USA). Results were expressed as the signal ratio of GTPCH-1, DHFR, total eNOS to β-actin, or of eNOS dimer to eNOS monomer.

For quantitative RT-PCR, total RNA were isolated from mice lungs by using the EZNA total RNA kit (Omega Bio-Tek, Norcross, GA). Total RNA (1 µg) were reverse-transcribed by using the High-Capacity cDNA Reverse Transcription Kit (Applied Biosystems, Foster City, CA) according to the manufacturer’s instructions. Quantitative real-time RT-PCR amplification was performed in an iCycler iQ System (Bio-Rad Laboratories, Munich, Germany) using the ABsolute QPCR SYBR Green Fluorescein Mix kit (Thermo Fischer Scientific, Surrey, UK). The comparative threshold cycle (Ct) method was used for relative mRNA quantification [24]. Gene expression was normalized to the house-keeping gene GAPDH, and the amount of target gene mRNA expression in each sample was expressed relative to that of the control [25]. Primers were 5’-CCT TCC GCT ACC AGC CAG A-3’ (forward), 5’-CAG AGA TCT TCA CTG CAT TGG CTA-3’ (reverse) for mouse eNOS, and 5’-CTC AAC TAC ATG GTC TAC ATG TTC CA-3’ (forward), 5’-CCA TTC TCG GCC TTG ACT GT-3’ for GAPDH.

### Wire myograph studies

Intrapulmonary arteries were mounted in a myograph as previously described [16,26]. Contraction to phenylephrine and relaxation to acetylcholine (after precontraction with 3.10^-6^ M phenylephrine) were evaluated in the presence or absence of sepiapterin (10^-4^ M; 60 min pre-incubation) or the NOS-synthase inhibitor N^ω^-nitro-L-arginine methyl ester (L-NAME, 3.10^-4^ M; 30 min pre-incubation).

### Morphometric analysis of pulmonary arterioles

Left lungs were fixed in 4% paraformaldehyde, and dehydrated in increasing grade of ethanol. After delipidation with xylene, lungs were embedded in paraffin and cut into transverse sections (4 µm) which were stained with hematoxylin, eosin and orcein. Pulmonary vascular remodeling was assessed by measuring the percentage of wall thickness of the arterioles. All arterioles from one section of each mouse being associated with bronchioli were analyzed for their external diameter, external and internal areas. The diameter was analyzed along four axes across the center of the vessels and only arterioles with diameters inferior to 100 µm were taken into account. The wall thickness of all arteries on each lung section from 4 to 7 mice was analyzed using Image J software. The percentage of wall thickness (% wall thickness) was calculated as [(external wall areas) – (internal wall areas)] x 100/ external wall areas. 

### Right ventricular systolic pressure and hypertrophy

Mice were anesthetised (pentobarbital, 50mg/kg i.p.) and after thoracotomy, a heparin-filled hypodermic needle connected to a polyethylene catheter was placed into the right ventricular cavity by direct puncture of the right ventricle. Right ventricular systolic pressure was measured by use of a fluid-filled force transducer. The weight ratio: right ventricle / (left ventricle + septum) was calculated to assess hypoxia-induced right ventricular hypertrophy.

### Drugs, reagents and antibodies

Acetylcholine, phenylephrine and L-NAME were purchased from Sigma (St Quentin-Fallavier, France), L-sepiapterin from Schircks Laboratories (Jona, Switzerland) and pentobarbital from Ceva (Libourne, France). Mouse monoclonal antibodies anti-eNOS and anti-DHFR were obtained from BD Transduction Laboratories (Le Pont de Claix, France), anti-GTPCH-1 from Santa Cruz Biotechnology (Heidelberg, Germany) and anti-β-actin from Sigma. Polyclonal goat anti mouse IgG peroxidase conjugated were purchased from Thermo Scientific (Illkirch, France).

### Data analysis

Data were expressed as mean ± SEM of n experiments. Statistical evaluation was performed by the non-parametric Mann–Whitney test for biopterin concentrations, protein expression, morphometric analysis and right ventricular systolic pressure. Concentration-response curves were compared using analysis of variance. Student’s t test was used to measure statistical differences among weight ratio right ventricle / (left ventricle + septum). Values were considered statistically significant with p<0.05.

## Results

### Hypoxia-induced increase in right ventricular systolic pressure and right ventricular hypertrophy

Right ventricular systolic pressure was significantly increased in WT mice exposed to hypoxia for 10, 21 or 40 days (Table *1*). Hearts from those mice also displayed a progressive right ventricular hypertrophy, as evidenced by a significant increase of the weight ratio right ventricle / (left ventricle + septum) (Table *1*). These modifications are known to be correlated with the development of pulmonary hypertension.

**Table 1:** Effect of chronic hypoxia on right ventricular systolic pressure and hypertrophy.

	**NX**	**HX 10d**	**HX 21d**	**HX 40d**
**RVSP (mm Hg)**	22.3 ± 3.4	33.2 ± 2.3 *	38.5 ± 4.2 *	35.8 ± 3.2 *
**RV/(LV+S)**	0.28 ± 0.01	0.37 ± 0.01 ***	0.44 ± 0.01 ***	0.49 ± 0.01 ***

Right ventricular systolic pressure (RVSP) and weight ratio right ventricle / (left ventricle + septum) (RV / (LV+S)) in wild-type mice exposed to normoxia (NX) or 10, 21 and 40 days of hypoxia (HX 10d, HX 21d and HX 40d, respectively). Results are expressed as mean ± SEM from 6 to 14 experiments for RVSP and from 41 to 56 experiments for RV / (LV+S). * p<0.05, *** p<0.001, compared to NX.

### BH_4_ and BH_2_ contents in lungs from hypoxic WT mice

**Figure 1:**
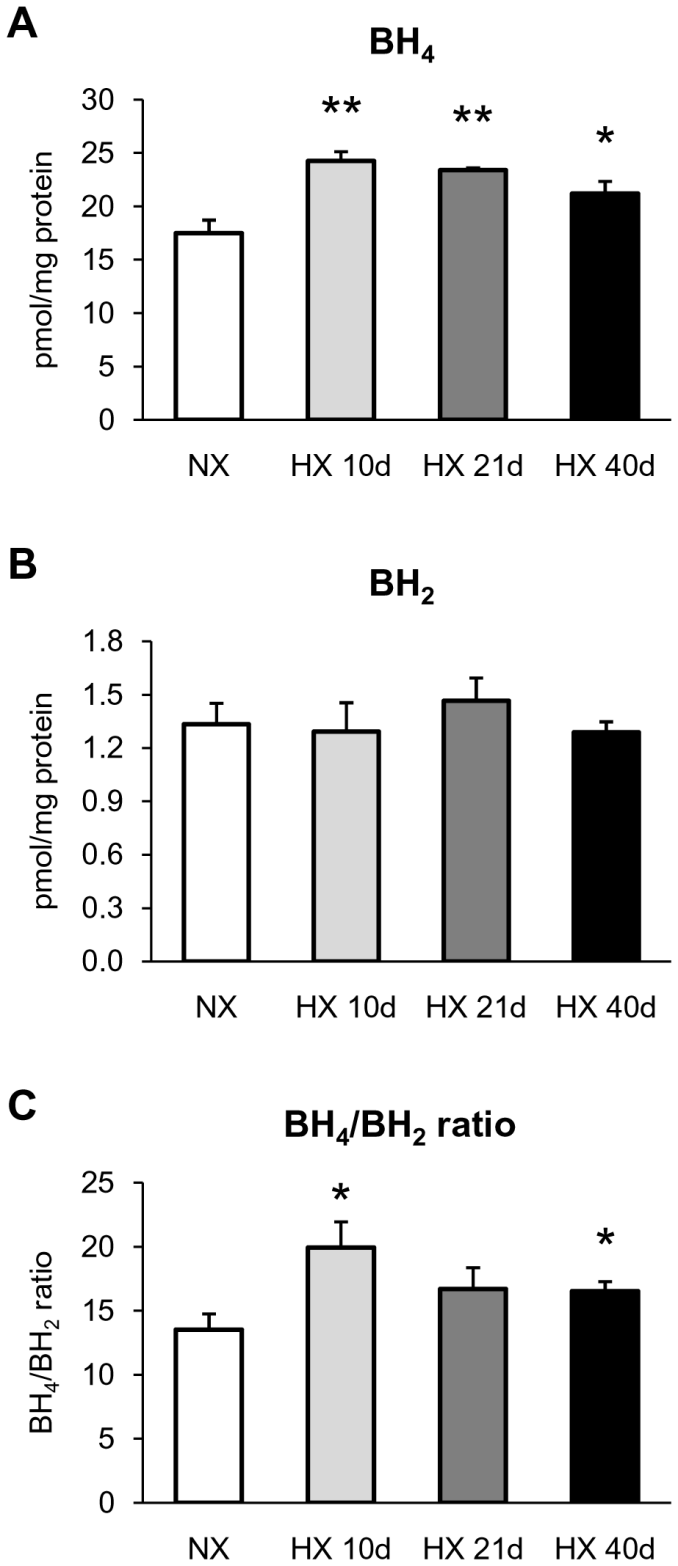
Effect of chronic hypoxia on lung levels of BH_4_ and BH_2_. (*A*) BH_4_ levels, (*B*) BH_2_ levels, and (*C*) BH_4_/BH_2_ ratio in lung from mice exposed to normoxia (NX) or 10, 21 and 40 days of hypoxia (HX 10d, HX 21d and HX 40d, respectively). Results are expressed as mean ± SEM from 6 experiments. * p<0.05, ** p<0.01, compared to NX.

BH_4_ levels significantly increased in lungs from WT mice exposed to hypoxia for 10, 21 or 40 days, compared to lungs from normoxic mice (Figure *1A*). Whatever the duration of hypoxia, BH_2_ levels in lungs remained stable compared to control lungs (Figure *1B*). Consequently, the BH_4_/BH_2_ ratio increased following chronic hypoxia, statistical significance being achieved at 10 and 40 days of chronic hypoxia, compared to normoxic mice.

### GTPCH-1, DHFR and eNOS expression in lungs and intrapulmonary arteries from hypoxic WT mice

**Figure 2:**
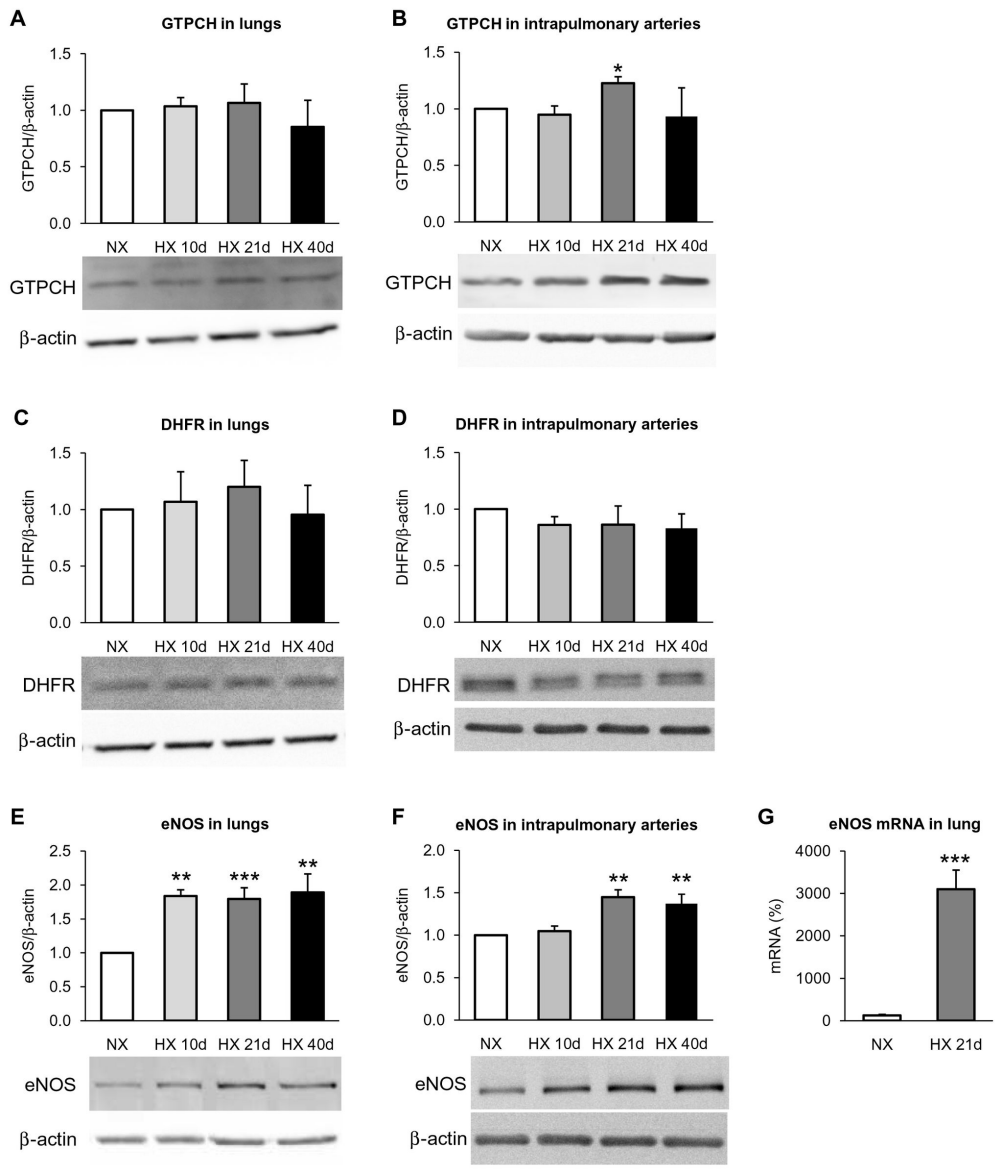
Effect of chronic hypoxia on expression of GTPCH-1, DHFR and eNOS in lungs and intrapulmonary arteries. Graphs showing quantified data normalized to β-actin and representative Western-blotting for (*A* and *B*) GTPCH-1, (*C* and *D*) DHFR, and (*E* and *F*) total eNOS, in lungs (ACE) and intrapulmonary arteries (BDF), from mice exposed to normoxia (NX) or 10, 21 and 40 days of hypoxia (HX 10d, HX 21d and HX 40d, respectively). (*G*) Expression of eNOS at the mRNA level by quantitative RT-PCR in lung from mice exposed to normoxia (NX) or 21 days of hypoxia (HX 21d). Results are expressed as mean ± SEM from 5 (Western-blotting) to 9 (RT-PCR) experiments. * p<0.05, ** p<0.01, *** p<0.001, compared to NX.

In lungs, protein expression of GTCPH-1 and DHFR remained unchanged in WT mice exposed to hypoxia for 10, 21 and 40 days, compared to normoxic controls (*Figure 2AC*). However, chronic hypoxia markedly up-regulated eNOS in lungs, at both protein (Figure *2E*) and mRNA levels (Figure *2G*). In intrapulmonary arteries, quite similar results were obtained (*Figure 2BDF*), except for GTPCH-1 expression which was slightly but transiently increased after 21 days of hypoxia, compared to normoxic controls (Figure *2B*) and eNOS whose expression was not increased after 10 days of hypoxia (Figure *2F*).

**Figure 3:**
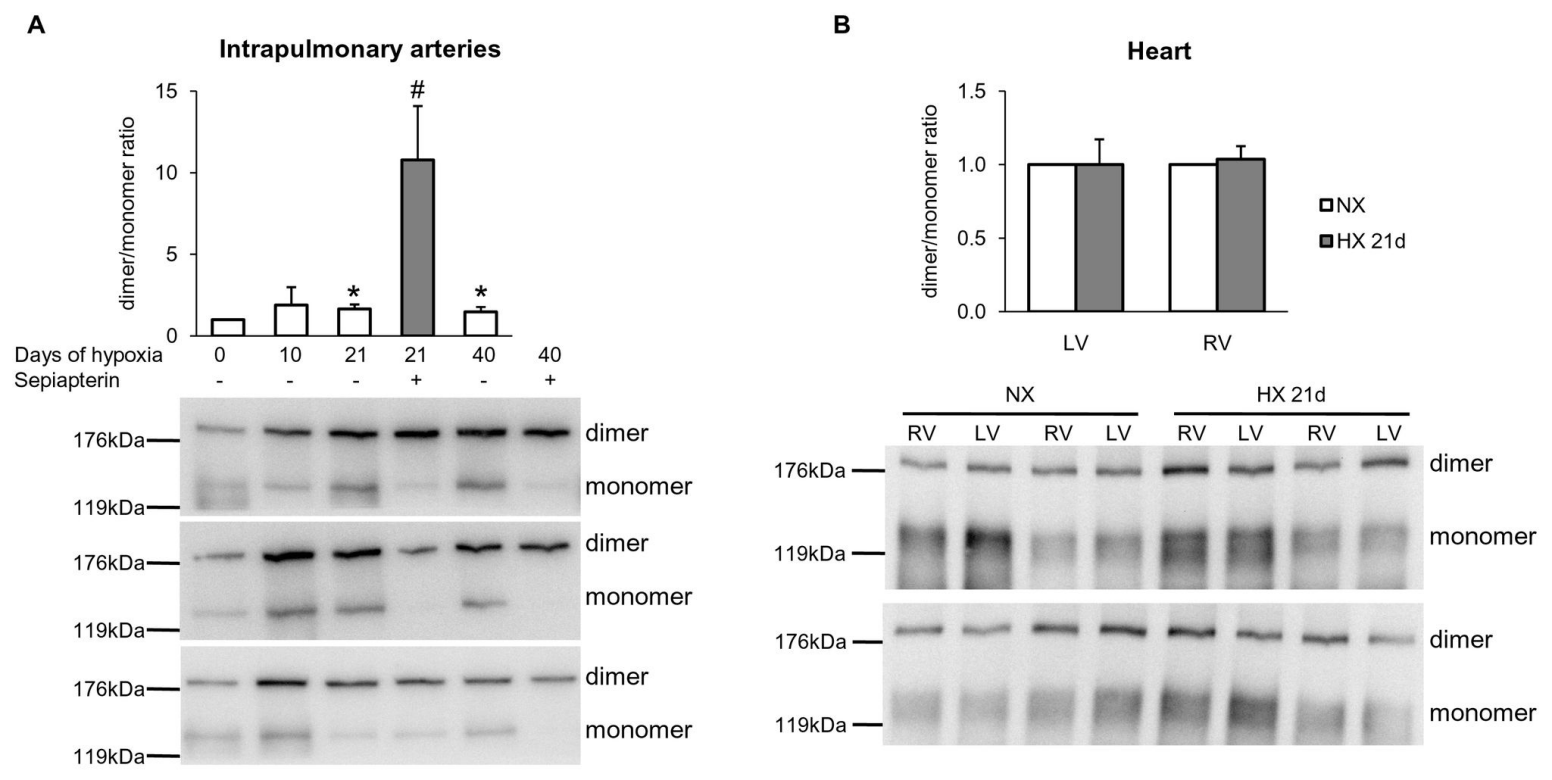
Effect of chronic hypoxia on eNOS dimer / monomer ratio in intrapulmonary arteries and cardiac ventricles. (*A*) Graphs showing quantified data for eNOS dimer / monomer ratio and representative Western-blots in intrapulmonary arteries from mice exposed to normoxia or 10, 21 and 40 days of hypoxia and treated or not with sepiapterin (10^-4^ M). (*B*) Graphs showing quantified data for eNOS dimer / monomer ratio and representative Western-blots in left ventricles (LV) and right ventricles (RV) from mice exposed to normoxia (NX) or 21 days of hypoxia (HX 21d). Results are expressed as mean ± SEM from 3 to 4 experiments. * p<0.05, compared to NX, ^#^ p<0.05 compared to 21 days hypoxia.

The dimer / monomer ratio of eNOS, which is an index of eNOS uncoupling, was investigated in intrapulmonary arteries of WT mice exposed to hypoxia. As shown in Figure *3A*, this ratio was unchanged after 10 days of hypoxia, but increased after 21 or 40 days of hypoxia (times at which BH_4_ content and total eNOS expression were both elevated), arguing against hypoxia-induced transition from eNOS dimers to eNOS monomers within the intrapulmonary vasculature. As expected, sepiapterin, a precursor of BH_4_, further enhanced eNOS dimerization in intrapulmonary arteries from hypoxic mice (Figure *3A*; after 40 days of hypoxia, the band corresponding to the monomer was even not detectable, not allowing accurate quantification of dimer / monomer ratio of eNOS). In right or left ventricles from normoxic or hypoxic (21 days) mice, no change in the dimer / monomer ratio of eNOS was evidenced (Figure *3B*).

### Effect of sepiapterin on hypoxia-induced alterations of pulmonary vasomotor responses, pulmonary vascular remodelling, right ventricular pressure and hypertrophy in WT mice

**Figure 4:**
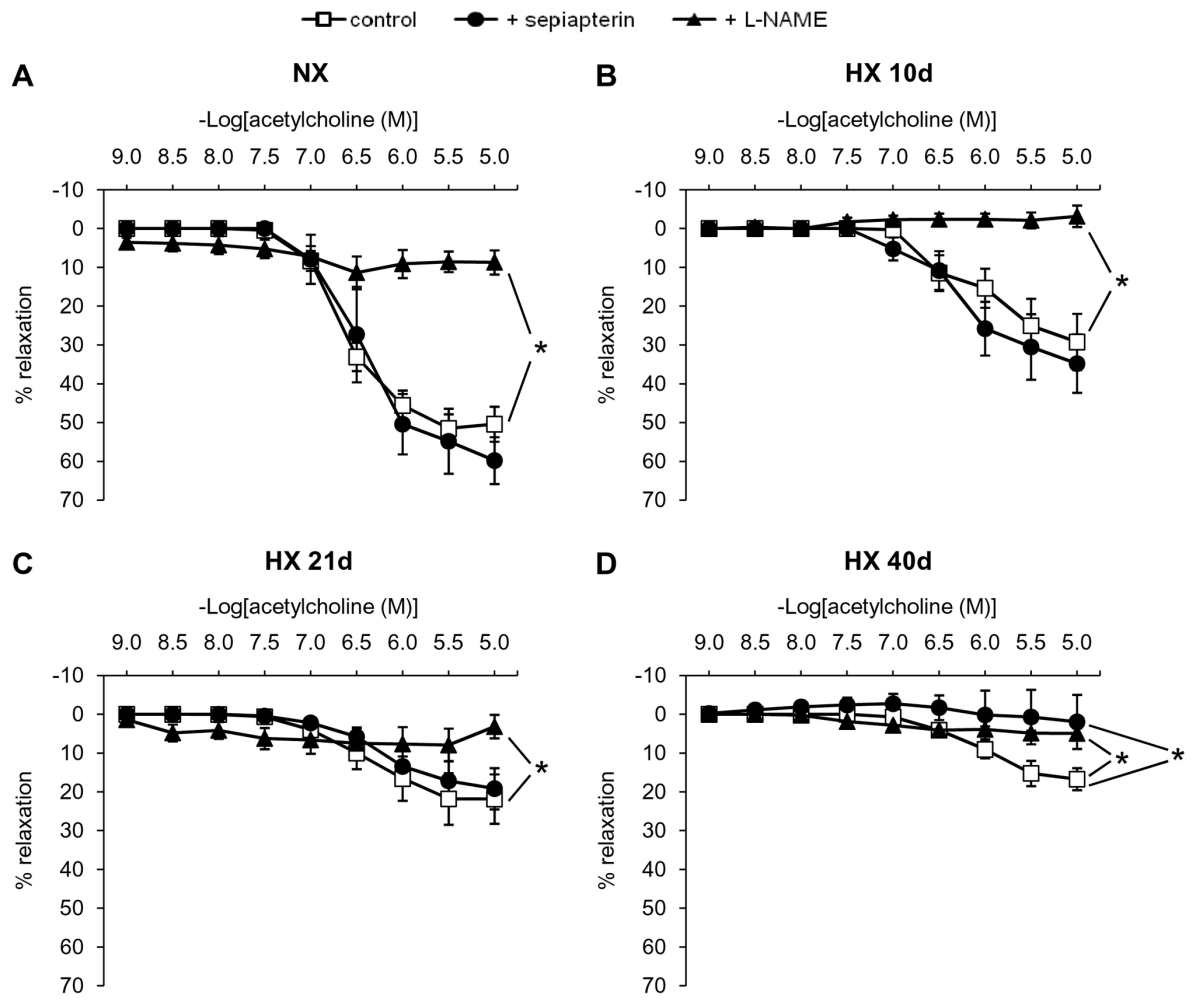
Effect of sepiapterin and L-NAME on hypoxia-induced alterations of relaxation to acetylcholine in intrapulmonary arteries. Relaxant response of acetylcholine in intrapulmonary arteries from mice exposed to (*A*) normoxia (NX), (*B*) 10 days, (*C*) 21 days, and (*D*) 40 days of hypoxia (HX 10d, HX 21d and HX 40d, respectively), in the presence or absence of sepiapterin (10^-4^ M) or L-NAME (3.10^-4^ M). Results are expressed as mean ± SEM from 4 to 6 experiments, 0% relaxation corresponding to the level of precontraction induced by 3.10^-6^ M phenylephrine * p<0.05 compared to control.

Endothelium-dependent relaxation to acetylcholine was progressively decreased in intrapulmonary arteries from WT mice exposed to hypoxia (for 10, 21 and 40 days), compared to normoxic controls (Figure *4*). In arteries from both normoxic and hypoxic mice, acetylcholine-induced relaxations were almost abolished by the NOS inhibitor L-NAME (Figure *4*), indicating that it was mediated by NOS-derived NO. Sepiapterin, in conditions in which it did not modify relaxation to acetylcholine in intrapulmonary arteries from normoxic mice but markedly increased dimer / monomer ratio of eNOS in intrapulmonary arteries from hypoxic mice (see Figure *3*), failed to normalize hypoxia-induced impaired relaxation to acetylcholine (Figure *4*). 

**Figure 5:**
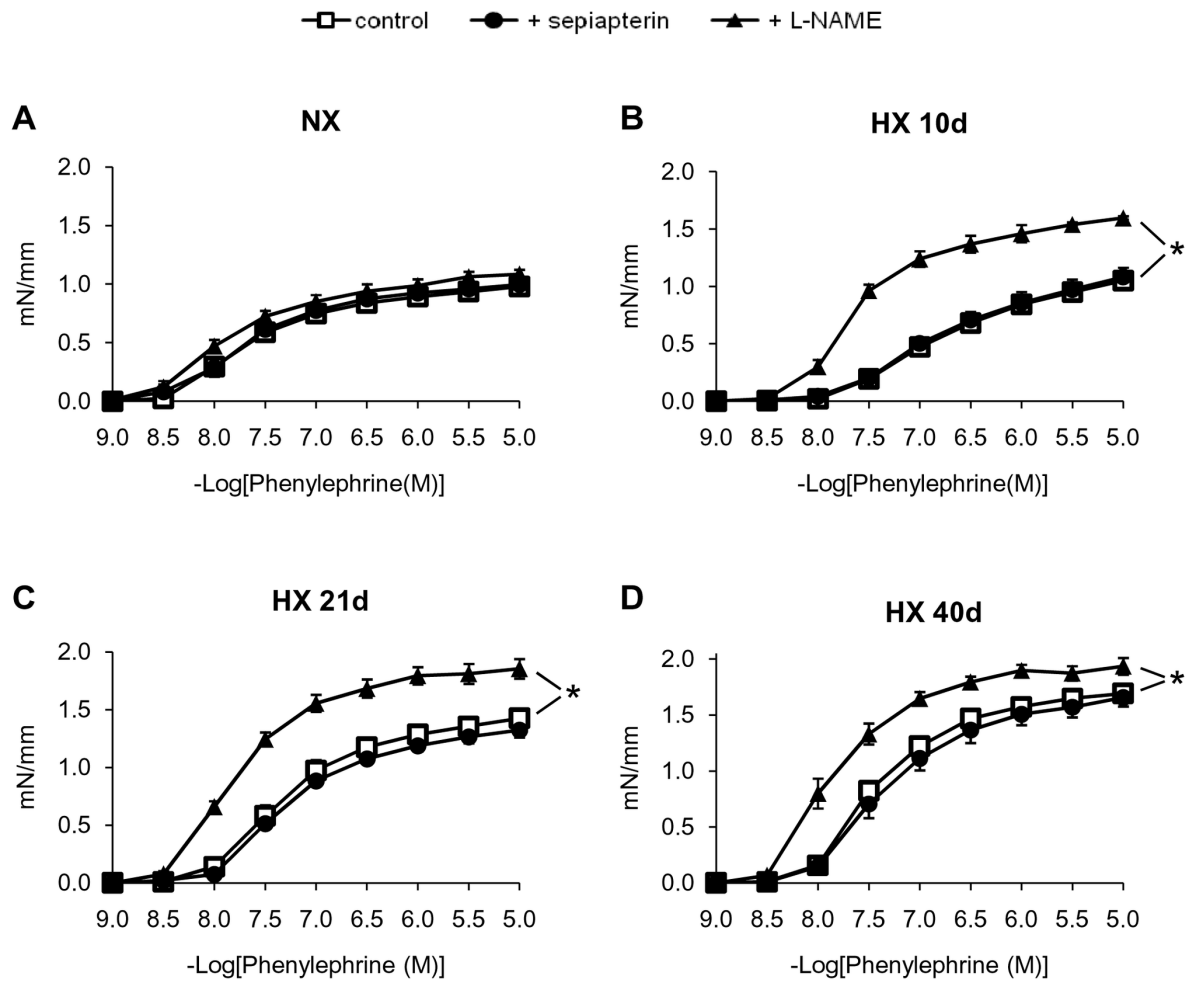
Effect of sepiapterin and L-NAME on hypoxia-induced alterations of contraction to phenylephrine in intrapulmonary arteries. Contractile response to phenylephrine in intrapulmonary arteries from mice exposed to (*A*) normoxia (NX), (*B*) 10 days, (*C*) 21 days, and (*D*) 40 days of hypoxia (HX 10d, HX 21d and HX 40d, respectively), in the presence or absence of sepiapterin (10^-4^ M) or L-NAME (3.10^-4^ M). Results are expressed as mean ± SEM from 6 to 9 experiments. * p<0.05 compared to control.

Phenylephrine-induced contraction was exacerbated following chronic hypoxia (Figure *5*). The NO-synthase inhibitor L-NAME exacerbated hypoxia-induced hyper-reactivity to phenylephrine in intrapulmonary arteries (Figure *5*), indicating that contractile responses were modulated by NOS-derived NO. Sepiapterin also failed to normalize hypoxia-induced impaired contraction to phenylephrine (Figure *5*). 

**Figure 6:**
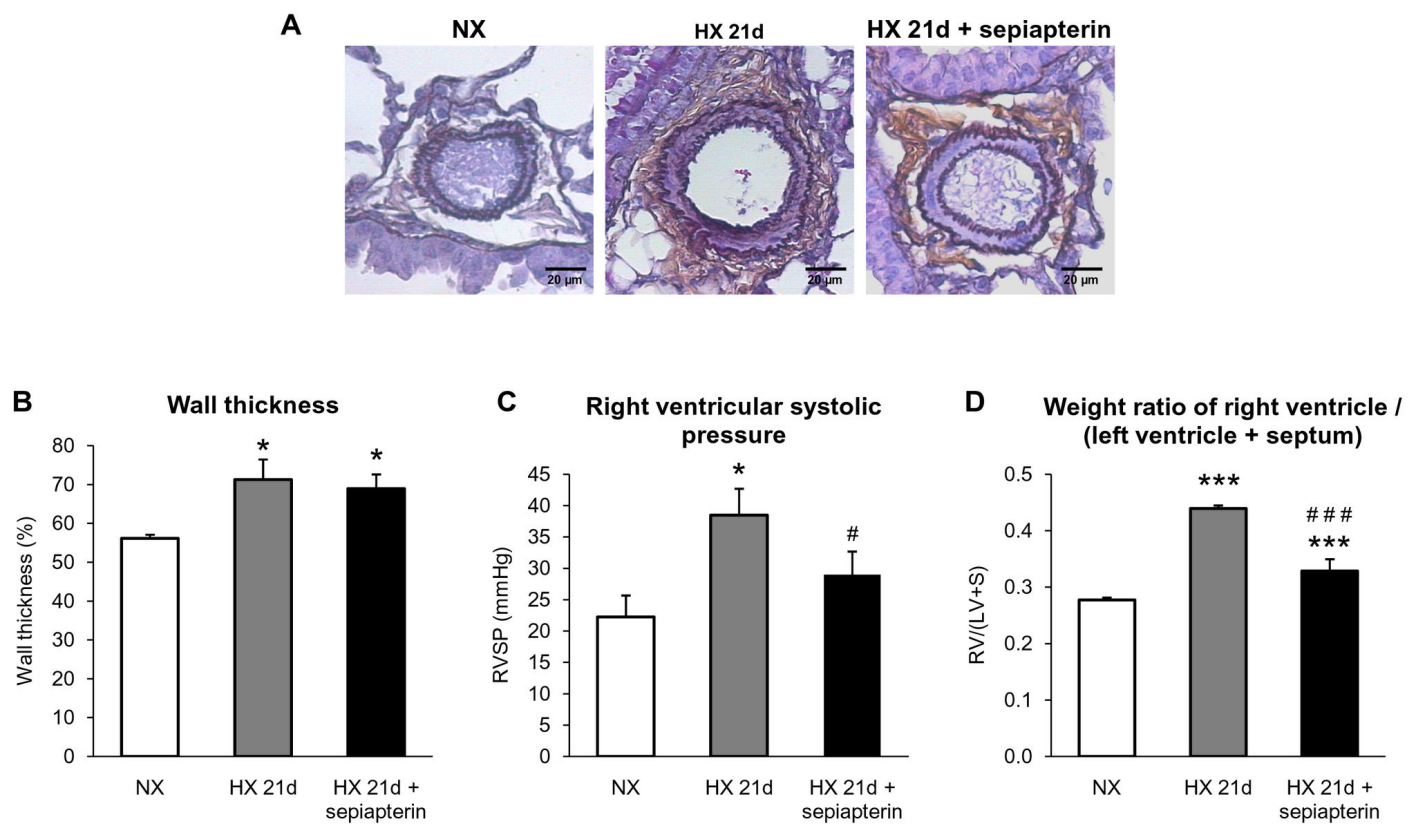
Effect of sepiapterin on hypoxia-induced remodeling of pulmonary arterioles, right ventricular pressure and hypertrophy. (*A*) Representative pictures, and (*B*) quantified data of wall thickness of pulmonary arterioles in mice exposed to normoxia (NX) or in mice exposed to 21 days of hypoxia (HX 21d) which were treated or not with sepiapterin (30 mg/kg, one day before exposure to hypoxia and every other day during hypoxia). (*C*) Right ventricular systolic pressure and (*D*) weight ratio right ventricle / (left ventricle + septum) in mice exposed to normoxia (NX) or in mice exposed to 21 days of hypoxia (HX 21d) which were treated or not with sepiapterin (30 mg/kg, one day before exposure to hypoxia and every other day during hypoxia). Results are expressed as mean ± SEM from all the arteries on each lung section from 4 to 7 mice for wall thickness, from 6-14 mice for right ventricular systolic pressure and from at least 8 experiments for weight ratio right ventricle / (left ventricle + septum). * p<0.05, *** p<0.001, compared to NX ; ^#^p<0.05, ^###^p<0.001 compared to 21 days hypoxia.

As expected, chronic hypoxia (21 days) significantly increased medial thickness in intrapulmonary arterioles (Figures *6A and B*), to a similar extend than in previous mice studies [27,28]. Chronic oral treatment with sepiapterin, wich tended to increase lung BH_4_ content from 23.4 ± 0.2 to 28.5 ± 2.4 pmol / mg protein (p = 0.10), did not prevent hypoxia-induced increase in pulmonary arterioles wall thickness (Figures *6A and B*). However, sepiapterin prevented hypoxia-induced increase in right ventricular systolic pressure (Figure *6C*) and right ventricular hypertrophy (Figure *6D*). In normoxic WT mice, both wall thickness of pulmonary arterioles and weight ratio right ventricle / (left ventricle + septum) were not modified by chronic oral treatment with sepiapterin (*data not shown*). 

### Pulmonary vasomotor responses, pulmonary vascular remodelling, right ventricular pressure and hypertrophy in hypoxic *eNOS* ^*-/-*^ mice

**Figure 7:**
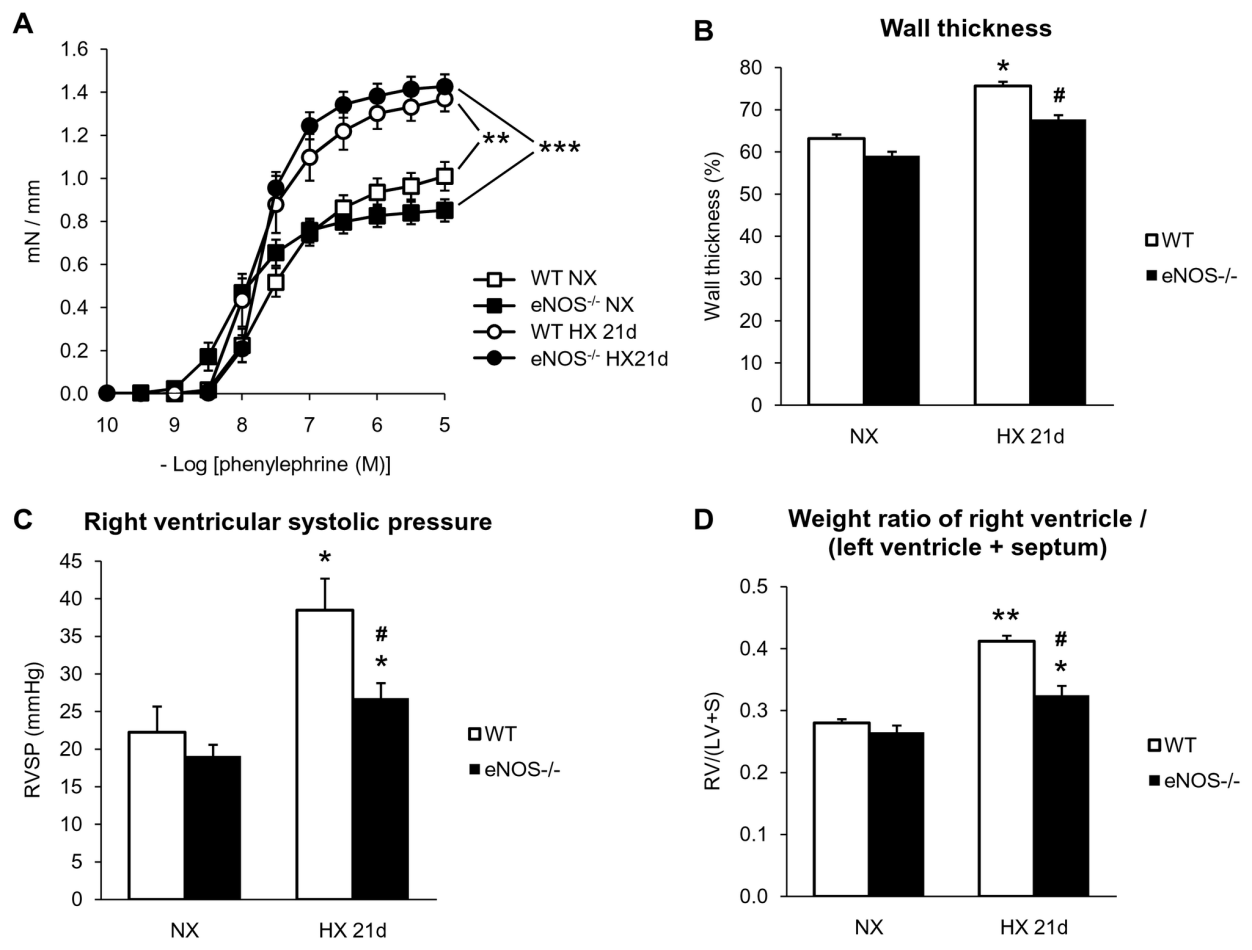
Effect of eNOS gene deletion on hypoxia-induced alterations of contraction to phenylephrine, remodeling of pulmonary arterioles, right ventricular pressure and hypertrophy. (*A*) Contractile responses to phenylephrine in intrapulmonary arteries, (*B*) wall thickness of pulmonary arterioles, (*C*) right ventricular systolic pressure and (*D*) weight ratio right ventricle / (left ventricle + septum) in wild-type (WT) and *eNOS* ^*-/-*^ mice exposed to normoxia (NX) or 21 days of hypoxia (HX 21d). Results are expressed as mean ± SEM from 8-18 experiments for reactivity to phenylephrine, 4-7 mice for wall thickness, 6-14 mice for right ventricular systolic pressure and from 41-94 experiments for weight ratio right ventricle / (left ventricle + septum). * p<0.05, ** p<0.01, *** p<0.001, compared to respective NX (WT or *eNOS* ^*-/-*^), ^#^ p<0.05 compared to WT exposed to 21 days of hypoxia.

As expected, eradication of eNOS gene markedly decreased relaxant responses to acetylcholine in intrapulmonary arteries (10 µM acetylcholine inducing 19.4 ± 2.7% and 50.4 ± 4.5% relaxation in *eNOS* ^*-/-*^ and WT normoxic mice, respectively). Residual relaxation to acetylcholine in intrapulmonary arteries from *eNOS* ^*-/-*^ mice was L-NAME insensitive and likely mediated by prostanoids and/or EDHF [26]. Deletion of eNOS gene did not prevent hypoxia-induced hyper-responsiveness to phenylephrine (Figure *7A*). However, following chronic hypoxia, remodelling of intrapulmonary arterioles (Figure *7B*), increase in right ventricular systolic pressure (Figure *7C*) and right ventricular hypertrophy (Figure *7D*) were all significantly attenuated in *eNOS* ^*-/-*^ mice, compared to WT (without any significant difference in body weight between these groups).

## Discussion

The main results of this study are the followings : (i)- in lungs, chronic hypoxia increased BH_4_ levels, without modifying BH_2_ levels and protein expression of GTPCH-1 and DHFR, two key enzymes regulating BH_4_ levels; (ii)- chronic hypoxia up-regulated eNOS expression in lungs and intrapulmonary arteries, but did not decrease eNOS dimer / monomer ratio in intrapulmonary arteries; (iii)- the BH_4_ precursor sepiapterin did not modify hypoxia-induced alterations of vasomotor responses or remodelling in intrapulmonary arteries, but it prevented increase in right ventricular systolic pressure and right ventricular hypertrophy; (iv)- eNOS gene deletion attenuated hypoxia-induced remodelling of pulmonary arterioles, increase in right ventricular systolic pressure and right ventricular hypertrophy. 

Data obtained in mutant models underline a pivotal role of BH_4_ in the pathogenesis of pulmonary hypertension [11,12]. We thus investigated BH_4_ level in a relevant pathophysiological mouse model of pulmonary hypertension. Since previous studies [11] demonstrated that, even though right ventricles from hypoxic mice displayed elevated systolic pressure and hypertrophy following one week hypoxia, lung level of BH_4_ remained unchanged, we determined lung BH_4_ levels after longer exposure to hypoxia (10, 21 and 40 days). We demonstrated that chronic hypoxia, whatever its duration, does not reduce the lung content of BH_4_, but rather increases it. The transient increase of GTPCH-1 expression (the limiting step for *de novo* BH_4_ biosynthesis) in intrapulmonary arteries following 21 days of hypoxia may contribute to BH_4_ content elevation in whole lung. However, no change of GTPCH-1 expression occurred after 10 or 40 days hypoxia, either in lungs or in intrapulmonary arteries, suggesting that increased catalytic activity of this enzyme, rather than increased expression, may be responsible for such increased BH_4_ content. 

In the absence of BH_4_ deficiency, increased BH_2_ levels (thus resulting in a decrease of BH_4_/BH_2_ ratio) is a determinant of eNOS uncoupling in intact cells [7-9]. We show here that the lung content of BH_2_ remained unchanged at the different time of hypoxia exposure, with no changes of DHFR protein expression (the enzyme which recycles BH_2_ into BH_4_). Together with the increase in BH_4_ level, the consequence is an increase and not a decrease, of the lung BH_4_/BH_2_ ratio. Modification of eNOS/BH_4_ stoicheiometry also induces eNOS uncoupling [10]. In accordance with previous studies [29,30], we show here that chronic hypoxia up-regulated eNOS expression (after 21 and 40 days of hypoxia in intrapulmonary arteries, and as early as 10 days and for subsequent duration of hypoxia in lungs). Thus, in lungs, time course of hypoxia-induced eNOS up-regulation and BH_4_ increase are similar, arguing against profound modification of eNOS/BH_4_ stoicheiometry. A decrease of the eNOS dimer / monomer ratio is generally accepted as an index of biopterin-dependent eNOS uncoupling [5]. Consistently with the increase in lung BH_4_, we show that chronic hypoxia increased, but did not decrease, eNOS dimer / monomer ratio in intrapulmonary arteries. This also argues against the role of another mechanism of eNOS dimers destabilization, biopterin-independent, such as the oxidation of the zinc-thiolate cluster of eNOS with subsequent release of zinc [31]. Altogether, these data demonstrate the absence of destabilization of eNOS dimers and biopterin-dependent eNOS uncoupling in the initiation and/or the progression of hypoxia-induced pulmonary hypertension.

The present study demonstrates that exogenous sepiapterin (a precursor of BH_4_ *via* DHFR) failed to modify hypoxia-induced remodelling and alteration in vasomotor responses of intrapulmonary arteries. This cannot be attributed to down-regulation of DHFR, since we showed that lung and intrapulmonary arteries expression of this enzyme remains unchanged following chronic hypoxia. We show here that chronic treatment with sepiapterin partially prevented hypoxia-induced increase in right ventricular systolic pressure and right ventricular hypertrophy. This cannot be attributed to prevention of hypoxia-induced eNOS destabilisation in cardiac tissue, since we show that chronic hypoxia did not modify dimer / monomer ratio of eNOS in right ventricles. The protective effect of sepiapterin on right ventricular systolic pressure and cardiac hypertrophy may be related to local increase in BH_4_, which may exert direct (i.e. NOS-independent) antioxidant effects [32,33]. As recently described, sepiapterin and BH_4_ protect soluble guanylate cyclase against oxidative inactivation, by a mechanism unrelated to NO synthase function and not limited to endothelial cells [34]. Sepiapterin also inhibits growth factors-induced cell proliferation and migration by a NO-independent mechanism [35]. Whether these mechanisms contribute to the cardiac protective effects of sepiapterin in hypoxic mice deserves future investigations.

In this study, some experiments were performed in *eNOS* ^*-/-*^ mice. The rationale was that some [36,37], but not all [38,39] studies, report that hypoxic *eNOS* ^*-/-*^ mice exhibit less remodelling of small pulmonary vessels or right cardiac ventricles, compared to hypoxic WT mice. An attractive hypothesis is that, in hypoxic WT mice, uncoupled eNOS generates deleterious ROS (promoting contraction and proliferation of pulmonary smooth muscle cells) rather than the vasculoprotective NO. We demonstrate here that, compared to hypoxic wild-type mice, hypoxic *eNOS* ^*-/-*^ mice display similar hyper-responsiveness to phenylephrine in intrapulmonary arteries, but are partially protected against hypoxia-induced remodelling of pulmonary arterioles, increase in right ventricular systolic pressure and right ventricle hypertrophy. The conflicting data regarding the consequence of eNOS deletion in pulmonary hypertension may depend on the genetic background of the mice [36], as exacerbation of remodelling was observed in mixed C57BL6/sv129 *eNOS* ^*-/-*^ mice [38,39], whereas attenuation was evidenced in C57Bl/6 *eNOS* ^*-/-*^ mice, as used here and previously [36,37]. Because BH_4_ and/or BH_2_-dependent eNOS destabilisation appears unlikely in cardiac ventricles and pulmonary vasculature from hypoxic mice, protection afforded by eNOS gene deletion might be explained by BH_4_ and/or BH_2_-independent eNOS uncoupling in WT hypoxic mice. These include L-arginine depletion [5], accumulation of the endogenous eNOS inhibitors methylarginines [5,40], defect in heat shock protein 90 / eNOS interaction [41], *S*-glutathionylation of eNOS [42] or thiylradical formation on the enzyme [43]. Even though some of these mechanisms are relevant in the pathogenesis of pulmonary hypertension [41,44-47], it remains unclear whether they necessarily imply modification of the dimer / monomer ratio of eNOS, which, as demonstrated here, was not affected in the pulmonary vasculature and right ventricles from hypoxic mice. Some compensatory mechanisms may also account for protection afforded by eNOS gene deletion. These may include activation of the cyclooxygenases-derived protective factors, which occurs in pulmonary vasculature of *eNOS* ^*-/-*^ mice [26] and/or activation of local atrial natriuretic peptide signalling, which prevents hypertensive cardiac hypertrophy in eNOS^-/-^ mice [48]. These hypotheses obviously deserve further investigations.

To conclude, this study demonstrates the absence of BH_4_/BH_2_ changes and eNOS monomerisation, which may induce eNOS uncoupling during hypoxia-induced pulmonary hypertension. Thus, even though eNOS gene deletion and sepiapterin treatment exert some protective effects, this appears unrelated to biopterin-dependent eNOS dimer destabilisation and uncoupling within pulmonary vasculature of hypoxic wild-type mice. Since right ventricular pressure and hypertrophy are clinical endpoints of pulmonary hypertension, the mechanisms of the cardioprotection afforded by eNOS gene deletion or sepiapterin treatment deserve to be further investigated.
